# Two Birds, One Stone: Double Hits on Tumor Growth and Lymphangiogenesis by Targeting Vascular Endothelial Growth Factor Receptor 3

**DOI:** 10.3390/cells8030270

**Published:** 2019-03-21

**Authors:** Ming-Chuan Hsu, Mei-Ren Pan, Wen-Chun Hung

**Affiliations:** 1 National Institute of Cancer Research, National Health Research Institutes, Tainan 704, Taiwan; mchsu@nhri.org.tw; 2 Institute of Clinical Medicine, College of Medicine, Kaohsiung Medical University, Kaohsiung 807, Taiwan; mrpan@cc.kmu.edu.tw; 3 Institute of Medicine, College of Medicine, Kaohsiung Medical University, Kaohsiung 807, Taiwan

**Keywords:** VEGF-C, VEGFR3, lymphangiogenesis, lymphatic metastasis

## Abstract

Vascular endothelial growth factor receptor 3 (VEGFR3) has been known for its involvement in tumor-associated lymphangiogenesis and lymphatic metastasis. The VEGFR3 signaling is stimulated by its main cognate ligand, vascular endothelial growth factor C (VEGF-C), which in turn promotes tumor progression. Activation of VEGF-C/VEGFR3 signaling in lymphatic endothelial cells (LECs) was shown to enhance the proliferation of LECs and the formation of lymphatic vessels, leading to increased lymphatic metastasis of tumor cells. In the past decade, the expression and pathological roles of VEGFR3 in tumor cells have been described. Moreover, the VEGF-C/VEGFR3 axis has been implicated in regulating immune tolerance and suppression. Therefore, the inhibition of the VEGF-C/VEGFR3 axis has emerged as an important therapeutic strategy for the treatment of cancer. In this review, we discuss the current findings related to VEGF-C/VEGFR3 signaling in cancer progression and recent advances in the development of therapeutic drugs targeting VEGF-C/VEGFR3.

## 1. Introduction

**Figure 1:**
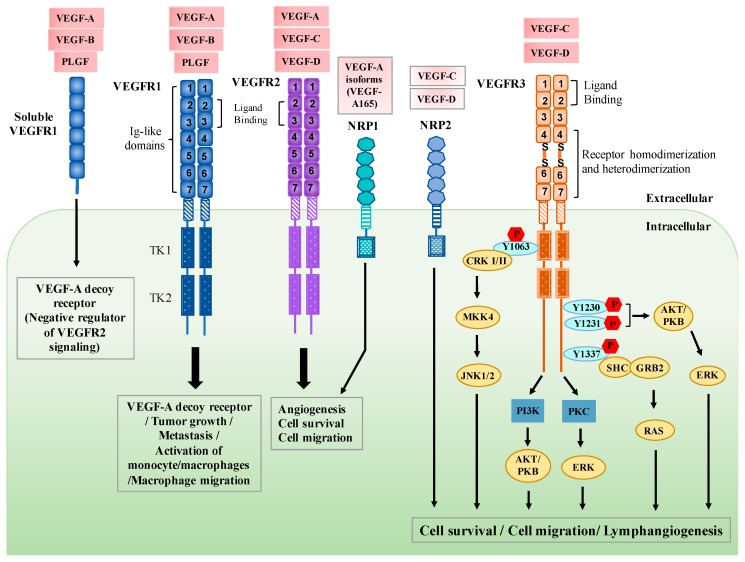
The signaling pathways of vascular endothelial growth factors and vascular endothelial growth factor receptors (VEGFs/VEGFRs) and their biological functions. The three tyrosine kinase (TK) receptors have specific binding capabilities. VEGF-A, VEGF-B, and PLGF can bind to VEGFR1 and mediate its biological functions. The binding of VEGF-A, VEGFR-C, and VEGF-D can stimulate the activation of VEGFR2, resulting in cell proliferation and angiogenesis. VEGF-C and VEGF-D bind to VEGFR3 and induce downstream signaling which mediates cell survival and lymphangiogenesis. Neuropilin 1 (NRP1) and neuropilin 2 (NRP2) can function as co-receptors for VEGFR2 and VEGFR3. The binding of VEGF-A isoforms and NRP1 can form a complex with VEGFR2, leading to the induction of downstream signaling which regulates the proliferation and migration of endothelial cells. VEGF-C/D bind to NRP2 and forms a complex with VEGFR3, activating the VEGFR3 signaling which enhances the proliferation of lymphatic endothelial cells (LECs) and lymphangiogenesis. MKK4, Mitogen-activated protein kinase kinase-4; JNK1/2, c-Jun N-terminal kinase-1/2; PI3K, phosphoinositide-3 kinase; AKT/PKB, AKT/protein kinase B; PKC, protein kinase C; ERK, extracellular signal–related kinase; SHC-GRB2, Src homology domain containing growth factor receptor–bound protein 2.

Vascular endothelial growth factor receptor (VEGFR) tyrosine kinases are critical regulators in the development and maintenance of blood and lymphatic vascular systems. In mammals, VEGFRs consist of three membrane proteins referred to as VEGFR1 (FLT1), VEGFR2 (KDR/FLK1), and VEGFR3 (FLT4) [1,2,3,4]. The activity of VEGFRs is modulated by five secreted glycoproteins, the vascular endothelial growth factors (VEGFs), which include VEGF-A, VEGF-B, VEGF-C, VEGF-D, and PLGF. The VEGF ligands bind to and activate three different VEGFRs, resulting in the stimulation of angiogenesis and lymphangiogenesis [5,6,7]. The *VEGFR1* gene produces two major proteins, a full-length receptor and a soluble VEGFR1 (sFlt-1). Full-length and soluble VEGFR1 are high-affinity receptors for VEGF-A, VEGF-B, and PLGF, and have been shown to function as negative regulators of VEGFR2 signaling [8,9,10,11]. In response to VEGF-A binding, VEGFR1 only exerts low activation of intracellular signaling and serves as a decoy receptor for VEGF-A, preventing its binding to VEGFR2 [12]. Although the kinase activity of VEGFR1 is relatively low compared with that of VEGFR2, the binding of PLGF can induce survival signals in endothelial cells and enhance angiogenesis [13]. In addition, several studies have shown that VEGFR1 signaling is critical for tumor growth, metastasis, activation of monocyte/macrophages, and macrophage migration [14,15,16,17,18]. VEGFR2 is another signaling receptor for VEGF-A and has been shown to play an important role in mediating vasculogenesis and angiogenesis [19,20,21]. VEGFR3 preferentially binds to VEGF-C and VEGF-D, and the ligand binding activates its downstream signaling pathways to regulate lymphatic development and function [22,23,24,25] (Figure 1). 

## 2. Regulation of VEGFR3 Signaling

VEGFRs consist of seven immunoglobulin-like (IG) domains that comprise the ligand-binding part, a single transmembrane domain, and a cytoplasmic tail which contains the split kinase domains for transducing growth factor signals. However, IG domains of VEGFR3 are different from that of other VEGFRs, where the fifth IG domain of VEGFR3 is cleaved and the two processed parts are held together through a disulfide bond [26] (Figure 1). The first and second IG domains of VEGFR3 are responsible for ligand binding, whereas the fourth to seventh IG domains are important for receptor homodimerization, heterodimerization (VEGFR2/VEGFR3), and receptor activation [27,28]. It has been known that VEGF-C and VEGF-D have a high affinity for VEGFR3. A previous study shows that VEGF-C is essential for sprouting of the first lymphatic vessels from embryonic veins. In *Vegfc−/−* mice, endothelial cells can commit to the lymphatic endothelial lineage but do not form lymphatic vessel sprouts from the embryonic veins [25]. In contrast, no defects in formation of lymphatic vessel sprouts from the embryonic veins were observed in *Vegf-d*-deficient mice [29]. However, one study demonstrates that endogenous Vegf-d in mice is dispensable for lymphangiogenesis during development, but its expression significantly contributes to lymphatic metastasis of tumors [30].

VEGF-C binding induces VEGFR3 dimerization and enhances the phosphorylation of tyrosine kinases in the cytoplasmic tail, resulting in the increase of downstream signaling. These phosphotyrosine residues then serve as docking sites for recruiting cytoplasmic signaling mediators that elicit diverse cellular responses such as cell proliferation, migration, and survival. Phosphorylated Tyr1337 has been proposed to be a binding site for the Src homology domain containing growth factor receptor–bound protein 2 (SHC-GRB2) complex, which activates the KRAS signaling pathway and regulates the transformation activity of VEGFR3 [31]. VEGF-C-induced phosphorylation of Tyr1230 and Tyr1231 stimulates the AKT/protein kinase B (AKT/PKB) and extracellular signal–related kinase (ERK) signaling pathways, contributing to proliferation, migration, and survival of lymphatic endothelial cells (LECs) [32,33]. Phosphorylation of Tyr1063 of VEGFR3 mediates cell survival by recruiting CRK I/II and inducing c-Jun N-terminal kinase-1/2 (JNK1/2) signaling via mitogen-activated protein kinase kinase-4 (MKK4) [33]. VEGFR3 phosphorylation also triggers phosphoinositide-3 kinase (PI3K)-dependent activation of AKT and protein kinase C (PKC)-dependent activation of ERK1/2 pathways. Stimulation of both signaling pathways promotes the proliferation of lymphatic endothelial cells [32] (Figure 1). 

The signaling via VEGFRs is also modulated through interactions with their coreceptors, such as neuropilin 1 (NRP1) and neuropilin 2 (NRP2). Originally, neuropilins were found to be expressed in the nervous and vascular systems and were identified as axonal guidance factors implicated in nerve development. NRP1 is mainly expressed in arteries, whereas NRP2 is expressed in veins and LECs [34,35]. It has been described that NRP1 specifically binds to VEGF-A isoforms such as VEGF-A165 and forms a complex with VEGFR2. The formation of VEGF-A165/NRP1/VEGFR2 complex induces VEGFR2 phosphorylation and downstream signaling, which regulates the proliferation and migration of endothelial cells [36,37]. In the vascular system, the expression of NRP2 and VEGFR3 is mainly in lymphatic vessels [38,39]. *Nrp2*-deficient mice show small lymphatic vessels and capillaries, which implies that the expression of NRP2 is critical for the development of lymphangiogenesis [38]. Although the mechanism of NRP2-mediated lymphangiogenesis remains unclear, increasing evidence suggests that NRP2 binds to VEGF-C/D and forms a complex with VEGFR3, thereby activating the VEGFR3 signaling which enhances the proliferation of lymphatic endothelial cells and lymphangiogenesis [40,41,42]. 

VEGFR3 is initially expressed in all vascular endothelial cells during embryogenesis and early postnatal development but later becomes restricted to LECs and certain fenestrated capillaries [43,44]. Since VEGFR3 expression is restricted to lymphatic vessels, it has been used as a marker for lymphatic vessels [45]. However, increasing evidence suggests that VEGFR3 is upregulated in blood vessels in some tumors and chronic wounds during active angiogenesis [46,47,48,49]. VEGFR3 has also been shown to be expressed in neuronal progenitors, osteoblasts, and macrophages [50,51,52]. Furthermore, recent studies have indicated that VEGFR3 expression is detected in different types of cancers and it contributes to tumor progression and lymphatic metastasis (Table 1) [53,54,55,56,57,58,59,60,61,62,63,64,65,66,67,68,69,70,71,72,73,74,75,76,77,78,79,80,81,82,83,84,85,86,87,88,89,90,91,92,93]. 

## 3. Functional Roles of VEGFR3 in Lymphatic Endothelial Cells

Lymphatic vessels are an integral part of the cardiovascular system, and are important for tissue fluid homeostasis, immune surveillance, and lipid absorption. The lymphatic vasculature collects extracellular fluids, proteins, lipids, and immune cells through lymphatic capillaries and drains lymph into pre-collector vessels that contain valves, ultimately transporting into the venous circulation [94,95]. Defective development of lymphatic vessels causes several disorders including vascular malformation, lymphoedema, and lymphangiectasia [96], whereas enhanced lymphangiogenesis is associated with tumor metastasis and tissue inflammation [97]. It has been shown that growth of lymphatic vessels occurs upon the exposure of LECs to VEGF-C-induced VEGFR3 signaling [25]. Available data support that VEGFR3 is critical for lymphatic vessel development. For example, VEGFR3 mutations identified in human and mice are known to cause lymphoedema [24,98,99]. Moreover, mice with *Vegfr3* deletion die at around E10.5 due to failure of cardiovascular development [100]. Furthermore, VEGF-C/VEGFR3 signaling is also implicated in modulating the remodeling and homeostasis of lymphatic vessels. A study of *Vegf-c*-deficient mice suggested that VEGF-C signaling was required for the migration of LECs and the formation of lymphatic vessel sprouts from embryonic veins [25]. A recent study shows that LECs of *Vegf-c*-deficient mouse embryos fail to detach from the cardinal vein and are unable to form the dorsal peripheral longitudinal lymphatic vessel (PLLV) and the ventral primordial thoracic duct (pTD), which results in lethality of mouse embryos [101]. Results obtained from genetically engineered animals further support the essential role of VEGF-C in lymphangiogenesis showing that depletion of the matrix-binding adapter protein CCBE1 reduces proteolytic processing of VEGF-C by protease A disintegrin and ADAMTS3 metalloprotease, resulting in the attenuation of the VEGFR3 signaling and lymphangiogenesis [102,103]. In addition, overexpression of VEGF-C induces the proliferation of LECs and hyperplasia of the lymphatic vasculature through VEGFR3 [104]. 

## 4. Clinical Significance of VEGF-C/VEGFR3 Expression in Tumors

**Figure 2:**
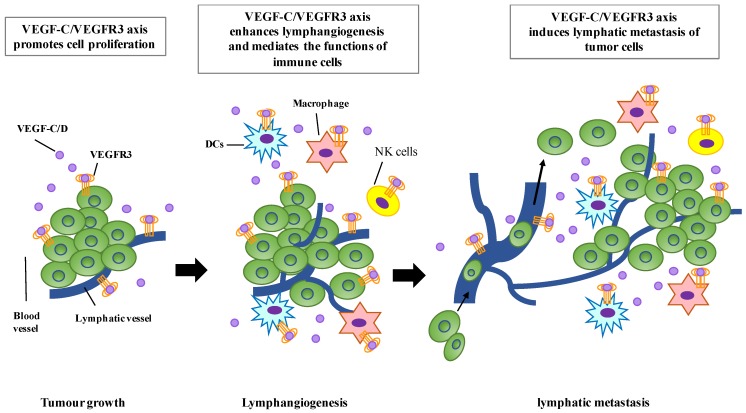
The function roles of VEGF-C/VEGFR3 signaling in tumor progression. The VEGF-C/VEGFR3 axis promotes tumor growth in autocrine and paracrine manners. VEGF-C can enhance the proliferation of LECs through VEGFR3, resulting in lymphangiogenesis and lymphatic metastasis of tumor cells. VEGF-C/VEGFR3 signaling can also mediate the functions of immune cells, including dendritic cells (DCs), macrophages, and natural killer (NK) cells.

Lymphangiogenesis is an important step in tumor progression. Dysregulation of lymphangiogenic factors has been known to promote lymphangiogenesis, which induces the formation of new lymphatic vessels that connect with the surrounding lymphatic vessels and provide routes for the transport of tumor cells to distant sites. The potential roles of the VEGFR-C/VEGFR3 axis in regulating tumor lymphangiogenesis and progression have been suggested. The expression of VEGF-C is detected in a variety of human tumors [105,106,107,108,109,110,111,112] and the increased level of VEGF-C is significantly correlated with lymph node metastasis, distant metastasis, and poor prognosis [97,113]. VEGF-C overexpression in breast cancer cells activates the VEGF-C/VEGFR3 axis in LECs and induces the formation of lymphatic vessels within and around tumors, resulting in enhanced tumor metastasis through lymphatic vessels [114,115] (Figure 2). In addition, mice bearing VEGF-C-overexpressing human breast carcinoma cells exhibited increased lymphangiogenesis and tumor metastasis via the lymphatic vessels [116]. Moreover, a soluble form of VEGFR-3, a potent inhibitor of VEGF-C/VEGF-D signaling, can inhibit lymphangiogenesis and suppress tumor metastasis [117]. 

VEGFR3 is primarily expressed in LECs, but is also expressed in non-endothelial cells, such as tumor cells (Table 1). Recently, Batsi et al. reported that the expression of VEGFR3 was detected in the nuclei of tumor cells and endothelial cells of tumor vessels in both primary urothelial bladder carcinoma and their recurrent tumors. However, the expression of VEGFR3 was not correlated with tumor grade and clinical stage [53]. Previous studies have also demonstrated that VEGFR3 protein was detected in breast cancer specimens. High expression levels of several angiogenesis-related proteins, including VEGFR3, are observed in patients with early-stage breast cancer and are associated with clinicopathological parameters and survival outcome [54]. It has been shown in a mouse model that the expression of VEGF-C and VEGFR3 promotes tumor growth and metastasis in an autocrine manner, whereas treatment with a VEGFR3 antagonist significantly suppresses tumor growth and lung metastasis [55]. Eroglu et al. also found that, while VEGFR3 is expressed in breast cancer cells, its expression is not associated with lymph node metastasis [56]. 

A recent study demonstrated that tumor-associated macrophages induced the expression of VEGF-C and VEGFR3 in lung adenocarcinoma cells, resulting in enhanced migration and invasion of cancer cells. Blockade of VEGFR3 signaling inhibits tumor growth and markedly suppresses the migration and invasion of tumor cells by upregulating the expression of p53 and PTEN. Furthermore, the study’s data revealed that the inhibition of VEGFR3 enhances chemosensitivity of doxorubicin in lung adenocarcinoma cells [58]. 

VEGFR3 expression has also been found in ovarian cancer cells and activation of the VEGFR3 signaling is induced by VEGF-C, which is produced by tumor-associated myeloid cells. The inhibition of VEGFR3 signaling results in the down-regulation of BRCA expression and cell cycle arrest. Moreover, VEGFR3 blockade chemosensitizes ovarian cancer to cisplatin chemotherapy in vitro and in vivo [59]. Decio et al. confirmed that VEGF-C and VEGFR3 were expressed in ovarian tumor tissues. VEGF-C released by tumor cells stimulates the VEGFR3 signaling in a paracrine and autocrine manner, leading to an increase in tumor growth and metastasis. Targeting the VEGF-C/VEGFR3 pathway decreases tumor burden and dissemination of ovarian tumors [60]. In renal cell cancer (RCC), the expression of VEGFR3 has been demonstrated in several studies [61,62]. Furthermore, Zhang et al. showed that VEGFR3 expression is correlated with histological grade, the status of lymph node, and metastasis in papillary renal cell carcinoma. Moreover, the expression of VEGFR3 can serve as a prognostic marker for papillary renal cell carcinoma and is also a predictor of lymph node metastasis as well [63]. 

Immunohistochemical analysis and qRT-PCR studies have demonstrated that the expression of VEGFR3 was increased in endometrial carcinomas compared with normal endometrium [64,65]. Additionally, VEGFR3 expression was significantly associated with tumor stage and poor disease-free survival in endometrial carcinomas [64]. Zhu et al. found that VEGFR3 was highly expressed in tissue samples of colorectal cancer. High expression of VEGFR3 was associated with the TNM (tumor, node, metastasis) stage and lymph node metastasis of colorectal cancer. The authors further illustrated that lipopolysaccharide (LPS) could upregulate VEGFR3 expression through increasing the binding of NF-κB to the promoter of VEGFR3, thereby promoting the migration and invasion of colorectal cancer cells [66]. Another study also showed that VEGFR3 expression was found in colorectal cancer and its expression was associated with lung metastasis [67]. 

A previous study showed that VEGFR3 expression was also found in gastric cancer and correlated with poorer prognosis, TNM stage, and lymphatic metastasis [69]. Recently, Dai et al. showed in an orthotopic mouse model that treatment with VEGFR3 antibody-conjugated ginsenoside Rg3 nano-emulsion might inhibit the expression of VEGF-C, VEGF-D, and VEGFR3, resulting in the suppression of tumor growth and lymphatic metastasis of human gastric cancer [70]. The expression of *VEGFR3* mRNA and protein were also detected in multiple cancers, including bladder, oral, head and neck, esophageal, and cervical cancers [71,72,73,74,75,76,77]. 

In prostate cancer, Yang et al. demonstrated that *VEGF-C* mRNA and VEGFR3 were highly expressed in tumorous prostate tissue. The expression of VEGFR3 is higher in *VEGF-C* mRNA-positive tumors compared to *VEGF-C* mRNA-negative tumor tissues. Thus, VEGFR3 expression is associated with poor prognosis and metastasis in human prostate cancer [79]. High expression levels of VEGFR2 and VEGFR3 were also detected in several medullary thyroid carcinoma (MTC) samples [80]. Another study investigated the influence of RAS mutation on the expression of TKI target proteins in MTC tumors. The results showed that VEGFR3 protein is expressed in few RAS-positive tumors and VEGF is frequently expressed in wild-type tumors. These findings could improve the selection of MTC patients for targeted therapy [81]. Kurenova et al. demonstrated that focal adhesion kinase (FAK) and VEGFR3 form a complex to promote cell proliferation in pancreatic ductal adenocarcinoma (PDA). They further showed that a small molecule inhibitor C4 could disrupt the interaction of FAK and VEGFR3 and inactivate FAK/VEGFR3 signaling to suppress cancer cell growth. Moreover, the combination of C4 and gemcitabine showed a significant synergistic effect on tumor suppression in PDA [82]. Another small molecule inhibitor C10 was also found to target the FAK/VEGFR3 complex and inhibit the growth of pancreatic tumor in vivo [83]. 

The expression of VEGFR3 has been detected in neuroblastoma cell lines, and the blockade of FAK-VEGFR3 interaction by C4 has also reduced cellular migration and proliferation. In addition, the combination of C4 and doxorubicin significantly suppressed tumor growth in a xenograft animal model [84,85]. VEGFR3 expression has been found in melanoma. Targeting FAK-VEGFR3 interaction by the small molecule C4 significantly inhibits melanoma tumor growth in vivo [87]. Recently, VEGF-C and VEGFR3 were found to be expressed in basal cell carcinoma (BCC). Yeh et al. demonstrated that the VEGF-C/VEGFR3 axis enhances the migration, invasion, and stemness of skin cancer cells via the KRAS/YAP1/Slug pathway. Targeting the VEGF-C/VEGFR3 axis by VEGFR3 blocking peptide significantly suppressed skin cancer progression [92]. 

## 5. Expression and Function of VEGFR3 in Immune Cells

Lymphatic vessels transport fluid, soluble antigens, and immune cells from peripheral tissues to draining lymph nodes (dLNs), where adaptive immunity and tolerance are modulated [118,119]. In addition to providing the routes for the trafficking of peripherally activated dendritic cells (DCs) into dLNs to activate immune response, lymphatic vessels also provide the routes for cellular egress leading to immune resolution [120,121]. It has been reported that lymphangiogenesis often occurs in chronic inflammatory tissues, including inflammatory bowel disease, chronic airway inflammation, and psoriasis [120,122,123]. VEGF-C and VEGFR3 are largely responsible for the development of lymphatic vessels. The pathogenic roles of VEGF-C and VEGFR3 in chronic inflammatory diseases and immune response have been well characterized in recent investigations. A previous study showed that the systemic inhibition of VEGFR3 increases the formation of inflammatory edema and inflammatory cell accumulation despite the inhibition of lymphangiogenesis in a Keratin 14 (K14)-VEGF-A transgenic (Tg) mouse model. Chronic delivery of VEGF-C or VEGF-D (which activates VEGFR3 signaling) into the skin of K14-VEGF-A mice significantly suppressed chronic skin inflammation, epidermal hyperplasia, and accumulation of CD8 cells. Similar results were also found by intracutaneous injection of recombinant VEGF-C156S mutant protein, a specific VEGFR3 ligand, which significantly reduced skin inflammation [121]. D’Alessio et al. demonstrated that increased lymphangiogenesis and lymphatic function reduced inflammatory bowel disease. The authors found that the VEGF-C/VEGFR3 signaling mediates “resolving” macrophage activation and mobilization in a STAT6-dependent manner, resulting in bacterial antigen clearance from the inflammatory area to the draining lymph nodes [120]. Furthermore, the expression of VEGFR3 in different immune cells has been reported. Hamrah et al. demonstrated the expression of VEGFR3 in corneal DCs and its up-regulation in inflammation. The authors further characterized that VEGFR3^+^ DCs are CD11c^+^CD45^+^CD11b^+^ and mostly major histocompatibility (MHC) class II^−^CD80^−^CD86^−^, which belong to immature DCs of the monocytic lineage [124]. In addition, Fernandez Pujol et al. reported that VEGFR3 is detected in immature DCs. In the presence of angiogenic growth factors, the immature DCs can differentiate into endothelial-like cells [125]. The expression of VEGF-C, VEGF-D, and VEGFR3 in tumor-associated macrophages (TAMs) has been shown in human cervical cancer [126]. The study indicates that VEGF-C/VEGFR3-expressing TAMs may play an important role in peritumoral lymphangiogenesis. Moreover, Su et al. also demonstrate that the VEGF-C/VEGFR3 axis is critical for macrophage infiltration in lung cancer, and VEGFR3-mediated macrophage infiltration may be involved in the radiosensitization of lung cancer [127]. More recently, Zhang et al. show that Gram-negative bacterial infection or LPS stimulation can elevate the expression of VEGFR3 and VEGF-C through TLR4-NF-kB signaling in macrophage, whereas VEGF-C ligation of VEGFR3 forms a negative feedback loop to inhibit TLR4-induced inflammatory responses. Their results represent a self-control mechanism to prevent uncontrolled inflammation in macrophages during bacterial infection [128]. The expression of VEGFR3 was also reported in natural killer (NK) cells. Lee et al. showed that the NK cells from acute myeloid leukemia (AML) express higher levels of VEGFR3 and lower levels of IFN-γ compared to the NK cells from healthy donors [93]. Moreover, increased lymphatic vessels and lymph drainage are correlated with tumor progression and tumor-associated lymphangiogenesis to enhance immune tolerance [129,130,131]. Emerging evidence also demonstrates that inflammatory lymphangiogenesis is correlated with graft rejection in renal and renal transplants [132,133]. Therefore, it is likely that VEGFR3 in immune cells might play complex roles in stimulation and resolution of immune response. 

## 6. Development of Drugs That Target VEGF-C/VEGFR3 Signaling

As mentioned previously, tumor-associated lymphangiogenesis plays a critical role in the mediation of tumor metastasis and has emerged as a novel target for cancer treatment [134,135]. Currently, multiple therapeutic strategies have been developed for targeting VEGF-C/VEGFR3 signaling, including (1) small molecule receptor tyrosine kinase inhibitors (TKIs) of VEGFR3; (2) monoclonal antibodies or receptor traps targeting VEGF-C; and (3) neutralizing antibodies or peptides that block the VEGFR3 signaling. 

### 6.1. Small Molecule TKIs of VEGFR3

**Figure 3:**
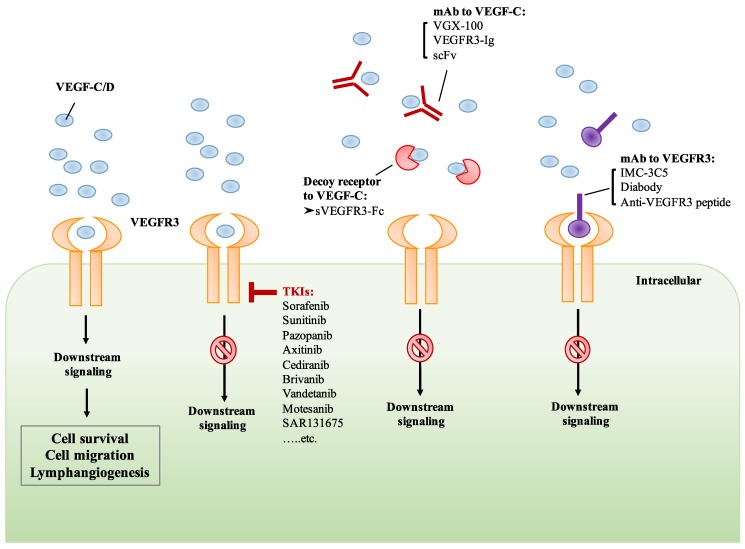
Targeting the VEGF-C/VEGFR3 axis by various therapeutic strategies. The binding of VEGF-C/D to VEGFR3 induces downstream signaling which mediates cell survival and lymphangiogenesis. The treatment of small molecule receptor tyrosine kinase inhibitors (TKIs) inhibits the activation of VEGF-C/D/VEGFR3 signaling. Monoclonal antibody (mAb) and receptor trap target VEGF-C, preventing its binding to VEGFR3. Monoclonal antibody or peptides targeting VEGFR3 prevent the binding of VEGF-C, resulting in the inhibition of VEGFR3 signaling.

Several TKIs have been developed for inhibiting the kinase activity of VEGFRs. Four TKIs that can be administered orally, namely, sorafenib, sunitinib, pazopanib, and axitinib, have been approved by the US Food and Drug Administration (FDA) and the European Medicines Agency (EMA) for clinical use [136,137] (Figure 3). The therapeutic efficacy of sorafenib monotherapy has been shown in patients with advanced renal cell carcinoma (RCC) and hepatocellular carcinoma (HCC) [138,139]. Sunitinib monotherapy has also shown significant improvement in progression-free survival (PFS) in patients with metastatic RCC [140]. The activity of pazopanib monotherapy was assessed in locally advanced or metastatic RCC, which showed improvement in PFS [141]. Recently, the therapeutic efficacy of axitinib has been demonstrated in metastatic renal cell carcinoma (mRCC) and the promising therapeutic efficacy of axitinib was demonstrated. Therefore, axitinib has been approved by the US FDA and EMA in the treatment of mRCC [137].

Cediranib is an oral VEGFR TKI and has been shown to suppress the activity of VEGFR2 and VEGFR3, leading to the inhibition of angiogenesis and lymphangiogenesis [142]. In the phase III ICON 6 trial, cediranib monotherapy has shown promising efficacy in platinum-sensitive relapsed ovarian cancer [143]. Brivanib, a selective dual inhibitor of VEGFRs and fibroblast growth factor receptors (FGFRs), has been evaluated in patients with advanced HCC. However, the results from phase III trials suggest that brivanib as an adjuvant therapy to transarterial chemoembolization (TACE) did not improve overall survival [144]. Moreover, the efficacy and safety of vandetanib in patients with advanced RET-rearranged non-small-cell lung cancer (NSCLC) was assessed in phase II trials. The clinical anti-tumor activity and a manageable safety profile of vandetanib were observed in patients with advanced RET-rearranged NSCLC [145,146]. Another TKI, motesanib, was tested in phase III trials in combination with paclitaxel and carboplatin (P/C) in advanced NSCLC patients. However, motesanib plus P/C did not significantly improve PFS [147] (Figure 3). Although the anti-tumor activity of TKIs has been reported, they are not highly selective since most of them target the ATP binding pocket. For example, sorafenib and sunitinib have been demonstrated to inhibit VEGFRs, platelet-derived growth factor receptors (PDGFRs), FGFRs, KIT, RET, and FLT3. These multi-targeted TKIs block a variety of kinases in addition to VEGFRs, resulting in adverse effects unrelated to VEGFR blockade. Therefore, the development of more specific VEGFR TKIs will improve anti-lymphangiogenic and anti-tumor activity with fewer off-target effects. Very recently, a small molecule TKI, SAR131675, has been reported to be highly specific for VEGFR3. The treatment of SAR131675 suppresses lymphangiogenesis and lymphatic metastasis in several experimental tumor models [148,149] (Figure 3).

### 6.2. Monoclonal Antibody Targeting VEGF-C/VEGFR3

Targeting the VEGF/VEGFRs signaling axis by using monoclonal antibodies has been demonstrated in recent years. The humanized anti-VEGF monoclonal antibody, bevacizumab, is an antibody approved by the US FDA for clinical use [150,151,152]. Bevacizumab-induced VEGF-A neutralization can prevent the binding of VEGF-A to VEGFR1 and VEGFR2, suppressing their activation and subsequent signaling cascades. Another neutralizing antibody against VEGFR2, ramucirumab, has also been approved for the treatment of various cancers including advanced gastric or gastro-esophageal junction adenocarcinoma, NSCLC, and advanced or metastatic urothelial carcinoma. Recently, a specific anti-VEGFR3 monoclonal antibody, IMC-3C5, has been assessed and has completed phase I trials in patients with advanced solid tumors and colorectal cancer (CRC). The results from the phase I study indicated that IMC-3C5 was well-tolerated up to the highest planned dose, but anti-tumor activity was not significant in CRC [153]. Another drug targeting the VEGF-C/VEGFR3 axis is VGX-100, a fully humanized VEGF-C neutralizing antibody which specifically binds to VEGF-C protein and thereby prevents its binding to VEGFR3. The therapeutic activity of VGX-100 was assessed in patients with advanced solid tumors in clinical phase I, and the trial was recently completed (ClinicalTrials.gov Identifier: NCT01514123) [154] (Figure 3). However, the results have not yet been published. 

Numerous antibodies, soluble receptor proteins, and IgG fusion proteins targeting the VEGF-C/VEGFR3 axis have been investigated in preclinical studies. Jimenez et al. developed a bispecific antibody which binds to both VEGFR2 and VEGFR3 in a dose-dependent manner and inhibits the interaction of VEGF-A/VEGFR2 and VEGF-C/VEGFR3. Their results showed a simultaneous dual blockade of VEGFR2 and VEGFR3 by the antibody, subsequently inhibiting the migration of endothelial cells [155]. A previous study demonstrated that a soluble VEGFR3 decoy receptor, sVEGFR3-Fc, expressed by a recombinant adeno-associated viral vector, potently suppressed tumor-associated lymphangiogenesis and lymphatic metastasis in highly metastatic melanoma, renal cell carcinoma, and prostate cancer models [156]. By using antibody phage-display, Rinderknecht et al. developed a human monoclonal antibody fragment (single-chain fragment variable, scFv) that specifically binds to VEGF-C with high affinity and inhibits VEGF-C/VEGFR3 signaling [157]. A new receptor-immunoglobulin (Ig) fusion protein, VEGFR3-Ig, that could simultaneously bind to VEGF-A and VEGF-C has been reported recently. VEGFR3-Ig has been shown to block tumor-associated angiogenesis, lymphangiogenesis, and metastasis in a highly metastatic HCC model [158]. In addition, Yeh et al. showed that VEGF-C/VEGFR3-mediated KRAS/YAP1/Slug pathway could be suppressed by treatment with anti-VEGFR3 peptide, leading to the inhibition of migration, invasion, and stemness of skin cancer cells [92] (Figure 3).

## 7. Conclusions

The VEGF-C/VEGFR3 axis has been implicated in cancer progression by directly affecting tumor cells or modulating lymphangiogenesis and immune response. High expression of VEGF-C/VEGFR3 has been demonstrated to be correlated with increased lymphatic metastasis and poor prognosis in numerous types of cancers (Table 1). Over the last two decades, tumor-associated lymphangiogenesis is considered as a potential target for treating metastatic diseases. Therefore, the development of drugs targeting the VEGF-C/VEGFR3 signaling has received much attention, which could be beneficial for patients with VEGF-C/VEGFR3-driven cancers. Multiple VEGFR TKIs have been tested in clinical/preclinical studies, and several VEGFR TKIs have been approved for clinical use (Table 2). However, these agents might inhibit multiple kinases in addition to VEGFR3, and the “off-target” effects might increase adverse effects. Hence, development of more selective and specific anti-VEGFR3 TKIs is required. In addition, the VEGF-C/VEGFR3 signaling has been shown to be involved in regulating immune tolerance and suppression [93,120,121]. Targeting the VEGF-C/VEGFR3 axis could enhance anti-tumor immune responses. Currently, several studies focused on VEGF-C/VEGFR3-mediated immunobiology in LECs and immune cells are now growing. The results from these studies will increase our understanding of how the VEGF-C/VEGFR3 axis affects immunity and will provide the rationale for the development of new immunotherapeutic strategies for cancer therapy.

## Figures and Tables

**Table 1:** Expression of VEGFR3 in tumor cells.

Tumor Type	Detection	Expression	Correlate to Lymph Angiogenesis	Correlate to Lymph Node Metastasis	Ref.
Urothelial cancer	IHC	Tumor cells	−	−	[53]
Breast cancer	IHC, RT-PCR, Western blot	Tumor cells	−	+	[54,55,56]
Lung cancer	IHC, qRT-PCR, Western blot	Tumor cells	−	+	[57,58]
Ovarian cancer	qRT-PCR, IHC, Western blot	Tumor cells	+	+	[59,60]
Renal cell cancer	IHC	Tumor cells	−	+	[61,62,63]
Endometrial cancer	IHC, qRT-PCR, Western blot	Tumor cells	−	+	[64,65]
Colorectal cancer	IHC, qRT-PCR, Western blot	Tumor cells	+	+	[66,67,68]
Gastric cancer	IHC, qRT-PCR	Tumor cells	+	+	[69,70]
Bladder cancer	Western blot	Tumor cells	−	−	[71]
Oral cancer	IHC	Tumor cells	+	+	[72]
Head and neck cancer	qRT-PCR, qMSP-PCR	Tumor cells	−	−	[73]
Esophageal cancer	IHC	Tumor cells	−	−	[74,75]
Cervical cancer	IHC, in situ hybridization, qRT-PCR, Western blot	Tumor cells	+	+	[76,77]
Prostate cancer	IHC, in situ hybridization, qRT-PCR	Tumor cells	+	+	[78,79]
Thyroid cancer	IHC	Tumor cells	−	−	[80,81]
Pancreatic cancer	Western blot	Tumor cells	+	+	[82,83]
Neuroblastoma	RT-PCR, Western blot	Tumor cells	−	−	[84,85]
Melanoma	Western blot, IHC	Tumor cells	+	−	[86,87]
Glioblastoma	In situ hybridization, qRT-PCR	Tumor cells	−	−	[88,89]
Osteosarcoma	IHC	Tumor cells	−	−	[90]
Laryngeal cancer	RT-PCR	Tumor cells	+	−	[91]
Basal cell carcinoma	qRT-PCR, Western blot	Tumor cells	−	+	[92]
Acute myeloid leukemia	RT-PCR, IHC	Acute myeloid leukemia (AML) natural killer (NK) cells	−	−	[93]

+, the expression of VEGFR3 is correlated with angiogenesis or lymph node metastasis; −, the expression of VEGFR3 is not correlated with angiogenesis or lymph node metastasis.

**Table 2:** Therapeutic agents for the inhibition of VEGF-C/VEGFR3 signaling.

Agents	Agent Description	Developer	Current Status	Ref.
Sorafenib	Small molecule TKI (VEGFRs, PDGFRs, c-kit, RET)	Bayer and Onyx	FDA-approved	[138]
Sunitinib	Small molecule TKI (VEGFRs, PDGFRs, c-kit, Flt3, RET)	Pfizer Inc.	FDA-approved	[140]
Pazopanib	Small molecule TKI (VEGFRs, PDGFRs, c-kit)	GlaxoSmithKline	FDA-approved	[141]
Axitinib	Small molecule TKI (VEGFRs, PDGFRs, c-kit)	Pfizer Inc.	FDA-approved	[142,143]
Cediranib	Small molecule TKI (VEGFRs, PDGFRs, c-kit)	AstraZeneca	Phase III	[137]
Brivanib	Small molecule TKI (VEGFRs, PDGFRs, FGFRs)	Bristol-Myers Squibb	Phase III	[144]
Vandetanib	Small molecule TKI (VEGFRs, PDGFRs, EGFR, RET)	AstraZeneca	Phase II	[145,146]
Motesanib	Small molecule TKI (VEGFRs, PDGFRs, c-kit, RET)	Amgen	Phase III	[147]
SAR131675	Small molecule TKI (more selective for VEGFR3 than VEGFR1/2)	Sanofi	Preclinical	[148,149]
Bevacizumab	Humanized anti-VEGF-A mAb	Genentech	FDA-approved	[150]
IMC-3C5	Humanized anti-VEGFR3 mAb	ImClone Systems/Eli Lilly	Phase I	[153]
VGX-100	Humanized anti-VEGF-C mAb	Circadian Technologies	Phase I	[154]
Diabody	Anti-VEGFR2/ VEGFR3 mAb	-	Preclinical	[155]
sVEGFR3-Fc	Soluble VEGFR3 decoy receptor	-	Preclinical	[156]
Single-chain fragment (scFv)	Anti-VEGF-C mAb fragment	-	Preclinical	[157]
VEGFR3-Ig	Anti-VEGF-C/A Receptor-Ig fusion protein	-	Preclinical	[158]
Anti-VEGFR3 peptide	Anti-VEGFR3 peptide	-	Preclinical	[92]
